# Influence of emergence delirium-related knowledge and nursing stress, practice, and confidence levels on the performance of recovery room nurses: A cross-sectional study

**DOI:** 10.1371/journal.pone.0314575

**Published:** 2024-12-09

**Authors:** Woo Jeong Ban, Jung Min Lee, Soo-Hyun Nam

**Affiliations:** 1 Kangwon National University, Chuncheon, Gangwon-Do, South Korea; 2 School of Nursing, Hallym University, Chuncheon, Gangwon-Do, South Korea; 3 Department of Nursing, Andong National University, Andong, South Korea; University Magna Graecia of Catanzaro, ITALY

## Abstract

In this study, we aimed to explore the relationships between knowledge of emergence delirium (ED) and nursing stress, practice, confidence, and performance levels, as well as to identify factors influencing the performance of recovery room nurses. We conducted a cross-sectional study with 135 recovery room nurses at a general hospital in South Korea. The nurses completed a questionnaire from April 12 to April 25, 2023. Data were analyzed using multiple linear regression to examine factors associated with nursing performance in relation to ED. Nursing performance showed a significant negative correlation with nursing stress levels and a moderate positive correlation with nursing practice and confidence levels. Furthermore, ED education, nursing practice, and nursing confidence are factors influencing nursing performance. To enhance the performance of recovery room nurses in caring for patients with ED, we recommend developing a tailored educational program that focuses on increasing both the confidence levels and practical skills of nurses, thereby meeting the specific needs of the patients.

## Introduction

Emergence delirium (ED)—a medical emergency characterized by cognitive and behavioral disturbances—causes symptoms including confusion, restlessness, aggressive behavior, and physiological responses such as elevated blood pressure and heart rate during recovery from anesthesia [1–4]. The 2017 European Society of Anesthesiologists guidelines define ED as postoperative delirium that occurs from the immediate end of anesthesia up to 24 h afterward [5]. ED is also known as emergence agitation or post-anesthetic delirium and affects approximately 87%, 50%, 37–46%, and 10–24% of critically ill patients, older adults, the general population undergoing anesthesia during surgery, and the general adult patient population with medical conditions, respectively [6]. The causes of ED are multifactorial, involving physiological, pharmacological, and psychological factors [7]. Although the exact mechanisms underlying ED are not fully understood, potential contributors include the type and dosage of anesthetic agents, patient age and pre-existing medical conditions, surgical procedure, and pain and discomfort experienced during recovery [4].

ED is a serious and costly medical condition that is recognized as a significant indicator of job-related stress among nurses. Moreover, it causes short-term discomfort and distress, thereby potentially disrupting the recovery process, delaying discharge from the post-anesthesia care unit (PACU), and increasing healthcare costs [8]. Furthermore, ED places severe stress on medical staff, particularly nurses who provide urgent care for patients with ED symptoms [9]. Patients with ED may become violent, unstable, and uncooperative, leading to potential injury, worsening of their condition, and delays in performing other tasks. This creates a stressful environment for nurses, especially when patients become violent, as nurses must balance their fear with their responsibility to ensure patient safety. Consequently, work-related stress associated with ED is likely to increase [9]. Work-related stress can directly and indirectly impact the provision of healthcare services and nursing performance. Such stress can adversely affect employee morale and confidence, work performance, productivity, and overall quality of life, which ultimately exerts a negative impact on patient outcomes [10–12].

ED frequently occurs during the postoperative period. Nurses working in the PACU, recovery rooms, or surgical units can promptly detect subtle changes in patient condition after routine assessments [5,7]. However, owing to a lack of ED-related knowledge and experience, nurses frequently miss the signs of ED. Consequently, behavioral symptoms of ED are often mistakenly attributed to personality traits, anxiety, depression, or psychosis [13]. Over 50% of ED cases are under-recognized by nurses across various practice settings [5]. Therefore, adequate ED-related knowledge is crucial for its prompt identification and management, thereby enhancing direct patient care and reducing work-related stress caused by fear and ED-associated responsibility. Enhanced knowledge and nursing education on delirium have been shown to reduce the psychological strain on nurses caring for patients with delirium [5,14,15]. Delivering competent and safe patient care requires sound clinical judgment and a broad range of technical nursing skills. Nursing competence necessitates that nurses possess essential knowledge and skills when performing tasks, and a lack of knowledge can undermine nurses’ confidence levels and adversely affect their work performance [16].

Therefore, in this study, we aimed to assess ED-related knowledge levels among recovery room nurses to provide foundational data that would inform future ED-related educational strategies. Furthermore, we investigated the relationship of ED-related knowledge with nursing stress, practice, confidence, and performance, and the factors affecting the performance of recovery room nurses were identified. These data can serve as primary information for the future development of educational strategies and programs related to ED.

## Materials and methods

### Study design

This cross-sectional study employed a descriptive-correlational design to determine the relationship of ED-related knowledge with nursing stress, practice, confidence, and performance, and to identify influential factors related to the performance of recovery room nurses.

### Participants

The study included recovery room nurses across South Korea. The sample size was calculated using G*Power 3.1.9.4 for multiple linear regression with a fixed model, with R^2^ deviation from zero. The minimum sample size required was 129, assuming a significance level of 0.05, an intermediate effect size of 0.15, and a power of 0.95. We recruited 155 nurses to account for a potential dropout rate of 20%. Data were collected via an online survey conducted from April 12 to April 25, 2023. Nurses who met our inclusion criteria accessed the survey through an advertisement link posted on an online community where most nurses were registered. A total of 135 participants responded to the survey and agreed to participate in the study, resulting in a participation rate of 81.7%. No data indicated that the responses were negative.

### Ethical considerations

This study was evaluated and approved by the Institutional Review Board of HXX University (Approval number, 2XXX-01X; approval date, March 1, 2023). Ethical principles were strictly upheld, including anonymity, informed consent, and data confidentiality. All participants provided written informed consent with the assurance that their anonymity would be maintained. Participation was voluntary, and the data were stored in compliance with data protection regulations.

### Measures

#### ED-related knowledge

ED-related knowledge was assessed using the Emergence Delirium Knowledge Scale developed by Jung et al. [17]. The scale includes 34 items, with each correct and incorct response scoring 1 and 0 points, respectively. A higher score indicates greater ED-related knowledge. In the current study, Cronbach’s α was 0.61, consistent with that reported by Jung et al. [17].

#### ED-related nursing stress

ED-related nursing stress was measured using the Strain of Care for Delirium Index, originally developed by Vermeersch [18] and modified by Milisen et al. [19]. This scale was adapted into Korean by Jung et al. [17]. It consists of 22 items rated on a 5-point Likert scale from “very severe” (5 points) to “never” (1 point). Higher scores indicate greater ED-related nursing stress. In this study, Cronbach’s α was 0.91, aligning with the findings of Jung et al. [17].

#### ED-related nursing practice

To assess ED-related nursing practice, this study utilized a 4-point Likert scale developed by Koo et al. [20] and further modified by Jo et al. [21]. This tool includes 33 items, each rated from “always done” (5 points), “often done” (4 points), “sometimes done” (3 points), “rarely done” (2 points), to “) to “never done” (1 point). A higher score indicates better ED-related nursing performance. Cronbach’s α was 0.90 in the study by Jo et al. [21] and 0.91 in the present study.

#### ED-related nursing confidence

The Self-Confidence in Caring for Patients with Delirium Tool, developed by Akechi et al.[22], was used to measure nurses’ confidence in delirium management. This scale was translated and revised by Kim [23]. It comprises 15 items scored from 0 to 100 points, with higher average scores indicating greater nursing confidence in managing patients with delirium. In this study, Cronbach’s α was 0.96, consistent with that reported by Kim [23].

#### Nursing performance

Nursing performance was evaluated using a tool developed by Kim [23]. This instrument includes 17 questions across four factors: 7, 4, 3, and 3 questions assess nursing performance ability, nursing attitude, nursing work improvements, and nursing process application, respectively. Measurements were obtained via a 4-point scale, where higher scores indicate better nursing performance. Cronbach’s α was 0.92 in the study by Ko et al. [24] and 0.96 in the present study, which was consistent with that reported by Kim [23].

#### Other variables

The general and work-related characteristics of the participants included age, sex, marital status, religion, level of education, total clinical experience, and average recovery room experience. The following ED-related characteristics were also included: ED-related knowledge level, experience with ED training/education, ED training needs, necessary parts of ED training, and the workload required in caring for patients with ED.

#### Analysis

Data were analyzed using SPSS (version 27.0, IBM Corp, Armonk, NY, USA). Participant general characteristics and ED-related knowledge as well as nursing stress, performance, and confidence levels were analyzed using descriptive statistics, such as frequency, percentage, average, and standard deviation. Inferential statistics such as the t-test for independent samples, one-way analysis of variance, and Scheffé’s test were used to determine differences in ED-related knowledge as well as nursing stress, performance, and confidence levels based on the general characteristics of recovery room nurses. Correlations of ED-related knowledge with nursing stress, performance, and confidence levels among recovery room nurses were calculated using Pearson’s correlation coefficient. Finally, the factors affecting nursing work performance were analyzed using multiple linear regression.

## Results

### Participant general and ED-related characteristics

Participant general characteristics are presented in Table 1. Most of the 135 recovery room nurses included in this study were women (*n* = 120, 88.9%) and unmarried (*n* = 103, 76.3%). The average age was 30.54 ± 5.64 years, and participants aged ≤30, 30–39, and ≥39 years accounted for >50% (*n* = 70, 51.9%), 36.3% (*n* = 49) and 11.9% (*n* = 16), respectively. Atheism was the most common belief (*n* = 89, 65.9%). In terms of education, 75.6% (*n* = 102) of the participants had a university degree.

**Table 1:** General characteristics and characteristics related to ED (*N* = 135).

Characteristic	Item	*N*	%	M ± SD (min -max)
Gender	Men	15	11.1	
	Women	120	88.9	
Age	<30	70	51.9	30.54 ±5.64 (22–50)
	30–39	49	36.3	
	>39	16	11.9	
Married	Married	32	23.7	
	Single	103	76.3	
Religion	Atheism	89	65.9	
	Christianity	22	16.3	
	Buddhism	10	7.4	
	Catholic	14	10.4	
Highest level of education	College graduation	22	16.3	
	University graduation	102	75.6	
	Graduate school	11	8.1 _	
Total clinical experience (months)	< 36 (<3 years)	25	18.5	87.31 ±69.04 (2–344)
	36–59 (3–5 years)	30	22.2	
	60–119 (5–10 years)	49	36.3	
	> 119 (˃10 years)	31	23.0	
Average recovery room experience (months)	< 36 (<3 years)	64	47.4	47.65 ±47.03 (1–236)
	36–59 (3–5 years)	30	22.2	
	60–119 (5–10 years)	31	23.0	
	>119 (˃10 years)	10	7.4 _	
Level of ED knowledge	Very or mostly familiar	63	46.7	
	Little or no knowledge	72	53.3	
Whether there is ED training/education	Received	72	53.3	
	Did not receive	63	46.7	
Method of receiving ED education (*n* = 72)	Through clinical word education	35	25.9	
	Through college	17	12.6	
	Others (refresher education, in-hospital education, academy, seminars, etc.)	20	14.8	
ED training needs	Very necessary	19	14.1 _	
	Necessary	50	37.0	
	Average need	14	10.4	
	Not necessary	26	19.3	
	Not very necessary	26	19.3	
Necessary parts of ED training	Prevention, assessment, and diagnosis	31	23.0	
	Arbitration and administration	104	77.0	
Degree of workload in providing care to patients with ED	Not difficult or normal	19	14.1	
	Slightly difficult	48	35.6	
	Very difficult	68	50.4	

M, mean; SD, standard deviation.

Regarding clinically relevant characteristics, the average clinical experience was 87.31 ± 69.04 months, with 5–10 years of experience being most common (*n* = 49, 36.3%), followed by 3–5 (*n* = 30, 22.2%), ≥10 (*n* = 31, 23.0%), and <3 years (*n* = 25, 18.5%). Most nurses had <3 years of recovery room experience (*n* = 64, 47.4%), followed by those with 5–10 (*n* = 31, 23.0%), 3–5 (*n* = 30, 22.2%), and ≥10 years (*n* = 10, 7.4%).

The majority of the participants (*n* = 72, 53.3%) reported having “little or no knowledge” of ED, whereas 63 (46.7%) participants described themselves as “very or generally well aware.” Seventy-two (53.3%) respondents stated they had ED training experience, and 63 (46.7%) had no experience. Among those who had received education, “through clinical experience” was the most common method (*n* = 35, 25.9%), followed by “refresher education, in-hospital education, academies, seminars” (*n* = 20, 14.8%), and “in college” (*n* = 17, 12.6%).

In terms of the need for ED education, most participants considered it “very necessary or necessary” (*n* = 69, 51.1%), followed by “not necessary” (*n* = 26, 19.3%), “very unnecessary” (*n* = 26, 19.3%), and “average” (*n* = 14, 10.4%). When asked about the essential aspects of ED education, “arbitration and administration” was the most frequently noted area (*n* = 104, 77.0%), with “prevention, assessment, and diagnosis” also highlighted (*n* = 31, 23.0%).

Regarding the workload required to provide nursing care to patients with delirium tremens, “very difficult” was the most common response (*n* = 68, 50.4%), followed by “slightly difficult” (*n* = 48, 35.6%), and “not difficult or normal” (*n* = 19, 14.1%).

### ED-related knowledge level

The overall ED-related knowledge level was 75.3%, with a mean score of 25.60 ± 3.83 (Table 2). The highest percentage of correct responses was for “patients can remove medical devices attached to their body” (98.5%, *n* = 133), followed by “patients can cry or struggle” 97.8% (*n* = 132) and “patients may remove invasive devices by themselves (e.g., A-line, C-line, chest tube, Jackson-Pratt drain),” with a correct response rate of 95.6% (*n* = 129). The question “ED delays departure from the recovery room” also received a high correct response rate. Conversely, the statement “ED is not related to changes in the surrounding environment” had a correct response rate of only 45.9% (*n* = 62), indicating confusion about the impact of environmental changes on ED. Similarly, “ED is a phenomenon that can occur when there is delayed emergence from anesthesia” had a correct response rate of 47.6% (*n* = 64). The item “If a patient experiences delirium, a restraint must be worn to prevent falls” also showed a correct response rate close to 50% (*n* = 67, 49.6%), highlighting areas where further education might be necessary.

**Table 2:** ED knowledge score (*N* = 135).

No.	Question	*N*(%)		Wrong question ranking
		Error	Hit	
1	It has nothing to do with the amount of intraoperative bleeding.	51(37.8)	84(62.2)	7
2	Delirium is likely to occur when the patient has problems with vision or hearing.	34(25.2)	101(74.8)	17
3	Emergence delirium may appear when the bladder is distended due to retention of the urine.	45(33.3)	90(66.7)	9
4	When emergence delirium develops, the patient’s prognosis may worsen.	36(26.7)	99(73.3)	13
5	There are tools to diagnose emergence delirium.	21(15.6)	114(84.4)	22
6	The patient may cry aloud or struggle.	3(2.2)	132(97.8)	33
7	Emergence delirium lasts a short time (usually about 30 minutes).	53(39.3)	82(60.7)	6
8	Emergence delirium is related to the patient’s level of consciousness prior to admission.	46(34.1)	89(65.9)	8
9	The possibility of emergence delirium increases if the patient has multiple diseases.	18(13.3)	117(86.7)	27
10	Slow speech, slow movement, and lethargy are not symptoms of emergence delirium.	41(30.5)	94(69.6)	11
11	Emergence delirium is associated with primary cerebral disorders (infection, stroke, trauma, etc.)	40(29.6)	95(70.4)	12
12	Emergence delirium has nothing to do with whether or not the patient has undergone surgery.	32(23.7)	103(76.3)	19
13	Patients may also remove the invasive device themselves. (A-line, C-line, Chest tube, JP drain)	6(4.4)	129(95.6)	31
14	Emergence delirium is related to hypoxia.	35(259)	100(74.1)	15
15	Emergence delirium is more likely to occur after prolonged surgery.	21(15.6)	114(84.4)	22
16	Emergence delirium delays the time to leave the recovery room.	6(4.4)	129(95.6)	31
17	The patient should be provided with a quiet and calm environment.	7(5.2)	128(94.8)	30
18	Emergence delirium is related to the patient’s psychiatric status before surgery.	43(31.9)	92(68.1)	10
19	Emergence delirium is related to the number of medications the patient is currently taking.	64(47.4)	71(52.6)	5
20	Emergence delirium has several different names.	34(25.2)	101(74.8)	17
21	Emergence delirium is more common in young people than in older people.	19(14.1)	116(85.9)	26
22	In the event of emergence delirium, the state of consciousness must be assessed.	15(11.1)	120(88.9)	29
23	Emergence delirium has nothing to do with changes in the surrounding environment.	73(54.1)	62(45.9)	One
24	When emergence delirium occurs, it is helpful to have the patient’s family with them.	22(16.3)	113(83.7)	21
25	Emergence delirium is not related to preoperative anxiety.	20(14.8)	115(85.2)	25
26	Patients can remove medical devices attached to their bodies.	2(1.5)	133(98.5)	34
27	Emergence delirium is associated with postoperative pain.	21(15.6)	114(84.4)	22
28	Emergence delirium is not related to electrolyte imbalance.	35(25.9)	100(74.1)	15
29	Emergence delirium is not related to the patient’s nutritional imbalance before surgery.	36(26.7)	99(73.3)	13
30	When a patient develops emergence delirium, a restraint must be used to prevent falls.	68(50.4)	67(49.6)	3
31	Administering benzodiazepines before surgery reduces the incidence of emergence delirium.	66(48.9)	69(51.1)	4
32	Emergence delirium increases the need for human resources and economic cost.	18(13.3)	117(86.7)	27
33	Dehydration is a predisposing factor for emergence delirium.	31(23.0)	104(77.0)	20
34	Emergence delirium is a phenomenon that can occur when emergence from anesthesia is delayed.	71(52.6)	64(47.4)	2

### ED-related knowledge and levels of nursing stress, practice, confidence, and performance

Table 3 outlines the levels of ED-related knowledge and nursing stress, practice, confidence, and performance among recovery room nurses. The mean scores for ED-related knowledge, nursing stress, nursing practice, nursing confidence, and nursing performance were 25.61 ± 3.83 (range, 13–32), 80.81 ± 12.10 (range, 47–107), 125.03 ± 16.89 (range, 83–165), 852.96 ± 266.00 (range, 150–1,480), and 56.11 ± 11.67 (range, 32–82), respectively.

**Table 3:** Mean score of main variables (*N* = 135).

Item (Scope)	Minimum	Max value	M ± SD
ED Knowledge	13	32	25.61 ±3.83
Nursing Stress	47	107	80.81 ±12.10
Nursing Practice	83	165	125.03 ±16.89
Nursing Confidence	150	1480	852.96 ±266.00
Nursing performance	32	82	56.11 ±11.67

M, mean; SD, standard deviation.

### Differences in nursing performance based on ED-related characteristics

The differences in nursing performance based on ED-related characteristics were as follows. Statistically significant differences in nursing work performance were observed based on ED-related knowledge levels (*t* = 6.52, *p* < 0.001) and whether participants had received ED training/education (*t* = 3.60, *p* < 0.001).

A significant difference in nursing work performance was noted among the 72 participants who had received ED education, depending on the educational method employed (*F* = 5.81, *p* = 0.005). Participants educated through clinical experience scored higher (60.0 ± 11.21) than those who received their education in college (51.8 ± 10.6). Similarly, the nursing performance of participants who had education through in-hospital education or academies (51.8 ± 10.6) was lower than that of participants who attended seminars (64.1 ± 11.3).

Significant differences were observed in terms of ED training needs (*F* = 44.3, *p* < 0.001) and the perceived need for ED training (*F* = 44.3, *p* < 0.001). Participants who described the training as “very necessary” (68.9 ± 7.74), “"needed” (65.4 ± 7.67), “very not needed” (48.8 ± 5.73), “not needed” (49.2 ± 7.88), and “normal” (49.7 ± 9.24) demonstrated varying levels of nursing performance. Additionally, a significant difference (*F* = 3.75, *p* = 0.026) was observed in nursing work performance based on the workload required in caring for patients with delirium tremens, and participants who reported “very difficult” conditions showed high nursing performance (58.27 ± 12.1).

### Correlations of ED-related knowledge with nursing stress, practice, confidence, and performance levels

Table 4 displays the correlations of ED-related knowledge with nursing stress, practice, confidence, and performance levels. In this study, nursing performance showed a significant negative correlation with nursing stress (r = -0.29, *p* = 0.001) and a moderate positive correlation with both nursing practice (r = 0.33, *p* < 0.001) and confidence (r = 0.31, *p* < 0.001) levels.

**Table 4:** Correlation between knowledge of ED, nursing stress, nursing practice, nursing confidence, and nursing performance (*N* = 135).

	r (*p)*				
	1	2	3	4	5
ED Knowledge (1)	.64				
Nursing Stress (2)	.26 (.003)	.91			
Nursing Practice (3)	.27 (.002)	.31 (< .001)	.91		
Nursing Confidence (4)	.31 (< .001)	0.25 (.004)	.36 (< .001)	.96	
Nursing Performance (5)	.12 (.176)	-.29 (.001)	.33 (< .001)	.31 (< .001)	.96

### Factors affecting nursing performance

Before conducting the multiple regression analysis, we confirmed that the assumptions for regression analysis were met, and the VIF value did not exceed 10, indicating the absence of multicollinearity. Additionally, the Durbin–Watson value was 2.09, falling within the 1.8–2.2 range, thus indicating no autocorrelation among residuals.

The variables that significantly impacted nursing performance included the perceived need for ED education (not necessary [*t* = 6.91, *p* < 0.001]; not very necessary [*t* = 7.28, *p* < 0.001]), nursing practice (*t* = 3.07, *p* = 0.003), and nursing confidence (*t* = 4.43, *p* < 0.001). The standardized coefficients suggest the relative impact of each variable on nursing performance, with the need for ED education showing the greatest influence (not very necessary [*β* = 0.53], not necessary [*β* = 0.48]), followed by nursing confidence (*β* = 0.25) and nursing practice (*β* = 0.17). The total explanatory power was 68.7% (R ^2^ = 0.72, Adju-R ^2^ = 0.69, *F* = 23.58, *p* < 0.001) (Table 5).

**Table 5:** Factors affecting nursing performance (*N* = 135).

Dependent variable	Independent variable		B	S E	*β*	T	*p*	VIF
Nursing Performance	(constant)		21.10	5.63		3.75	< .001	
	Level of knowledge of ED1)		- 2.79	1.86	-0.12	- 1.49	.136	2.74
	Whether there is ED training/education 2)		0.41	1.77	0.02	0.23	.817	2.48
	Method of receiving ED education 3)	Through clinical experience	-.92	2.07	-.035	-.44	.658	2.60
		Others (refresher education, in-hospital education, academy, seminars, etc.)	3.82	2.36	0.12	1.62	.108	2.23
	ED training needs 4)	Very necessary	0.47	1.81	0.01	0.24	.811	1.26
		Average	2.84	2.25	0.07	1.26	.210	1.49
		Not necessary	14.26	2.06	0.48	6.91	< .001	2.09
		Not very necessary	15.68	2.15	0.53	7.28	< .001	2.28
	Degree of workload in providing care to patients with ED 5)	Not difficult or normal	-2.38	1.99	-0.07	-1.19	.234	1.51
		Very hard	-0.07	1.35	-0.01	-0.05	.958	1.43
	Nursing Stress		0.07	0.06	0.078	1.34	.183	1.42
	Nursing Practice		0.12	0. 04	0.17	3.07	.003	1.33
	Nursing Confidence		0.01	0.01	0.25	4.43	< .001	1.32
R ^2^ = .72, Adj-R ^2^ = .69, F = 23.58, *p* < .001								

Reference groups: 1) little or no knowledge, 2) none, 3) currently in college, 4) necessary, and 5) somewhat difficult.

## Discussion

In this study, we aimed to investigate the relationships of ED-related knowledge with nursing stress, practice, confidence, and performance levels, and to identify influential factors related to the performance of recovery room nurses. Nursing performance demonstrated a significant negative correlation with nursing stress and a moderate positive correlation with both nursing practice and confidence. Moreover, the need for ED education, nursing practice, and nursing confidence were found to influence nursing performance. The main findings of the study are discussed below.

Overall, participant ED-related knowledge showed mixed results depending on the questions. Almost all participants correctly identified the manifestations (may cry or struggle to remove invasive and other medical devices from their bodies), interventions (providing quiet and calm environments), and implications (delay in leaving the recovery room) of ED. Our findings corroborated with the findings of a recent study by Igwe et al. [16] who reported that identifying signs of ED was not challenging. Nevertheless, Yuan et al. [5] reported difficulties in recognizing signs of ED, which was inconsistent with our study findings. However, over 50% of the participants incorrectly responded that ED is associated with environmental changes, ED can occur with delayed emergence from anesthesia, and patients experiencing ED should be restrained. Recovery room nurses should be able to accurately assess patients postoperatively to detect ED [7,9]. Our findings provide a valuable foundation for the development of educational resources on ED for recovery room nurses and can also serve as a basis for creating future nursing interventions or establishing clinical care guidelines for patients with ED. The implementation of educational interventions has been shown to significantly enhance the ability of nurses in delirium assessment and management. For example, a previous study demonstrated that the use of a delirium assessment training program effectively improved the skills of ICU nurses in delirium identification and management [25].

Statistically significant differences in nursing performance were observed among the participants. Nursing performance differed significantly according to the ED-related knowledge level, ED education experience, method of receiving ED education, perceived need for ED education, and the workload required in caring for patients with ED. Nurses who perceived a greater need for ED education demonstrated better nursing performance levels. Interestingly, our results revealed that nurses with little or no ED-related knowledge, those who received ED education through in-service training, and those with greater workloads had higher nursing performance levels. This finding contradicts findings of previous studies suggesting that experienced nurses with more knowledge of clinical practice generally demonstrate better work performance, productivity, and patient outcomes [10–12]. Our findings can be partially attributed to the participant characteristics, wherein the majority had at least a bachelor’s degree (83.7%), had <5 years of recovery room experience (69.6%), had not received ED education (53.3%), and perceived a challenging workload when caring for patients with ED (86%). Nurses with knowledge gaps and limited experience are often more conscious of their deficiencies. Thus, they tend to actively engage in programs and services to address their identified needs, which may eventually enhance their work performance [26].

Additionally, nurses who view ED as important are more likely to advocate for prompt interventions [5]. However, studies in Australia, Scotland, and Sweden have reported that the lack of established protocols and clinical guidelines on ED care may impact how nurses’ assessment and management of patients with ED [13,16]. Therefore, in addition to providing education and training to increase the awareness of nurses on ED and its importance, developing established protocols and clinical guidelines on ED at the hospital and institutional levels is essential. Our findings revealed statistically significant correlations between nursing performance, stress, practice, and confidence levels. Nursing performance was found to have a significant negative correlation with nursing stress and a moderate positive correlation with nursing practice and confidence. These findings align with those of Vermeersch et al. [18], who emphasized the psychological burden of ED-related stress on nursing competence. Similarly, a previous study revealed that managing delirium led to significant stress in ICU nurses, which in turn affected their job satisfaction and overall performance [27]. Stress management strategies should be implemented at work to enhance nursing performance; this will reduce nursing stress and alleviate the mental burden associated with nursing practice, thereby enabling nurses to approach their work with greater confidence. However, our findings revealed that ED-related knowledge was not significantly associated with nursing performance. This was inconsistent with the findings of Yuan et al. [5], who emphasized that nurses’ ED-related knowledge was crucial for initiating interventions and constituted a key aspect of nurses work performance.

Multiple regression analysis was conducted to determine the extent to which ED-related knowledge as well as nursing stress, practice, and confidence levels affected the performance of nurses caring for patients with ED. We found that the need for ED education, nursing practice, and nursing confidence influenced nursing performance. This result supports the findings of Milisen et al. [19], who reported that ED practice, confidence, and negative perceptions of the need for ED education were the strongest factors influencing the performance of nurses caring for patients with ED.

Extensive clinical experience in specialized settings, including recovery rooms, significantly enhances the knowledge and skills required to care for specific populations, such as patients with ED [20,21]. This can contribute to developing a highly specialized skill set that increases the confidence levels of nurses delivering patient care. Conversely, negative perceptions of the need for continuing or in-service education may adversely affect nursing competencies. Finally, the regression model revealed that nursing knowledge, stress, and confidence levels accounted for only 7.17% of the variance in the performance of recovery room nursing caring for patients with ED. Hence, multiple factors must be considered when assessing the performance of recovery room nurses caring for patients with ED.

Our findings highlight the importance of a structured educational approach in improving the performance of recovery room nurses caring for patients with ED. Incorporating specialized training modules focused on ED recognition and management into continuing education programs would be essential. Specifically, stimulation-based learning, may offer practical hands-on experience, thereby enhancing both the competence and confidence levels of nurses managing ED. These modules should emphasize real-life scenarios to better prepare nurses for the complexities of ED in PACU. Moreover, continuing education programs should include regular updates on ED-related knowledge, protocols, and management strategies to ensure that the nursing staff is well-equipped, ultimately improving patient outcomes.

This study has significant implications for nursing management and the development of interventions to support recovery room nurses. Nursing managers can use these findings to enhance the work environment by implementing stress reduction programs and providing continuous professional development opportunities. The negative effects of workplace stress on nursing performance can be alleviated by offering stress management workshops along with peer support systems. Furthermore, nursing performance can be boosted via the creation of an environment that promotes ongoing education and confidence-building through regular in-service training sessions. Nursing managers should also explore scheduling adjustments or workload balancing to reduce stress among recovery room nurses, thereby ensuring a healthier and more productive workforce.

Our study has several limitations. First, the use of self-reported measures introduces potential bias, such as social desirability or inaccurate self-assessment, which may affect the accuracy of the data on nursing stress, confidence, and performance levels. Second, the cross-sectional study design limits our ability to determine causality; thus, whether higher stress and lower confidence levels worsen nursing performance or vice versa remains unclear. Finally, the use of convenience sampling restricts the generalizability of the findings to all recovery room nurses, limiting a broader applicability of the results. A large-scale, randomized study should be conducted to provide more representative data.

Additionally, although this study focuses on a specific sample and region, future research should compare these findings with those from different healthcare settings or countries to assess consistency and broaden the understanding of the relationships of ED-related knowledge with nursing stress, confidence, and performance levels. Longitudinal studies would also be beneficial to track these factors over time, thereby providing deeper insights into their long-term effects and clarifying causal relationships. This would offer a more comprehensive understanding of how education and stress management can influence nursing performance in ED care.

## Conclusion

This study assessed the relationships between ED-related knowledge and nursing stress, practice, and confidence levels to evaluate the performance of recovery room nurses caring for patients with ED. ED-related knowledge was significantly associated with nursing stress, confidence, and practice levels. Although our findings indicated a relatively high level of ED-related knowledge among the participants, this was not directly associated with improved nursing performance. Instead, nurses with lower ED-related knowledge levels, more challenging workloads, and ED education obtained outside of their employment settings exhibited higher nursing performance. Nevertheless, the results of the individual items on the ED-related knowledge scale should be carefully reviewed and considered when developing continuing education programs to address knowledge gaps in ED care.
